# Neurolisteriosis in an Immunocompetent Adult Patient

**DOI:** 10.7759/cureus.98452

**Published:** 2025-12-04

**Authors:** Bivek Gurung, Ali Tarar, Paul Morris

**Affiliations:** 1 Internal Medicine, Doncaster and Bassetlaw Teaching Hospitals NHS Foundation Trust, Doncaster, GBR; 2 Diabetes and Endocrinology, Doncaster and Bassetlaw Teaching Hospitals NHS Foundation Trust, Doncaster, GBR; 3 Infectious Diseases, Doncaster and Bassetlaw Teaching Hospitals NHS Foundation Trust, Doncaster, GBR

**Keywords:** immunocompetent adults, listeria monocytogenes, meningitis, neurolisteriosis, rare cause of brain abscess

## Abstract

*Listeria monocytogenes* is an opportunistic pathogen that predominantly affects immunocompromised individuals, pregnant women, neonates, and the elderly. Here, we describe a rare case of meningitis caused by *Listeria monocytogenes* in an immunocompetent but otherwise healthy 30-year-old male. This was further complicated by a brain abscess and hydrocephalus, needing an external ventricular drain (EVD). This report outlines the diagnostic challenges encountered in general medicine, the treatment challenges, and the outcomes. Neurolisteriosis, though common in individuals with known risk factors, is nevertheless possible in immunocompetent, healthy patients. A high degree of suspicion and early treatment are important in patients who fail to respond to standard empirical antibiotics.

## Introduction

*Listeria monocytogenes* is an opportunistic, facultative, anaerobic, Gram-positive bacterium present ubiquitously in nature. *Listeria* infections include sepsis, meningitis, encephalitis, osteomyelitis, septic arthritis, spontaneous abortion, bacteraemia, and abscess formation. It commonly affects immunocompromised individuals, pregnant women, neonates, and the elderly. In a healthy immunocompetent adult, it can present with self-limiting gastroenteritis. There were 170 cases of listeriosis reported in England and Wales, with a crude incidence of 0.29 cases per 100,000 population in 2024, and the mortality rate among non-pregnant cases was 19.9% [1]. Pregnancy-associated cases were 20.75%, of which 43.7% resulted in stillbirth or miscarriage [1].

Atypical presentations of *Listeria *include invasive bacteraemia, endocarditis, and neurolisteriosis. Neurolisteriosis is an infection of the central nervous system (CNS). It commonly presents with meningitis or meningoencephalitis, but rhombencephalitis and brain abscesses are rare manifestations [2]. In this study, we present an atypical case of neurolisteriosis in an immunocompetent healthy individual. A key takeaway is the importance of maintaining a high degree of suspicion for neurolisteriosis and the need for early treatment in patients who do not respond to standard empirical antibiotics.

## Case presentation

A 30-year-old, previously healthy Caucasian man presented to the emergency department (ED) with headache, confusion, and blurred vision following a week of viral symptoms, extreme fatigue, dizziness, and vomiting. He had no travel history or significant medical conditions. Examination revealed an ataxic, unsteady gait and right eye diplopia. He was alert, with a Glasgow Coma Scale (GCS) of 15/15, with Eye (E) response 4, Verbal (V) response 5 and Motor (M) response 6 [3]. Pupils were equal and reactive, with normal ocular movements, and no nystagmus. No facial droop was observed, and he had an intact cranial nerve examination. Power and tone of all four limbs were normal with no sensory deficit. Normal reflexes and no cerebellar signs. No signs of meningeal irritation. Initial lab tests showed normal white cell count (WCC) 10.0 x 109/L (Normal 4.0 - 12.0 x 109/L), C-reactive protein (CRP) 1.58 mg/L (Normal 0 - 5), and erythrocyte sedimentation rate (ESR) 5 mm (Normal 1 - 10) (Table 1). Computerised tomography (CT) scans of the brain and venogram were normal. He was discharged for an outpatient magnetic resonance imaging (MRI) of the brain with contrast.

**Table 1:** Results of the blood investigation ESR: erythrocyte sedimentation rate; CRP: C-reactive protein; HCV AB: hepatitis C virus antibody; HBS AG: hepatitis B surface antigen; HIV: human immunodeficiency virus

Investigation	Day 0	Day 5	Normal Values	Unit
White Cell Count	10	14.6	4.0-12.0	x 10^9^ /L
Red Cell Count	5.41	5.73	4.4- 6.0	x 10^12^/L
Platelets	310	374	140-450	x 10^9^ /L
Haemoglobin	159	169	126-180	g/L
Neutrophils	6.1	13.6	2.0-7.5	x 10^9^ /L
Lymphocytes	2.9	0.5	1.5-4.0	x 10^9^ /L
Eosinophils	0.2	0	0.01-0.7	x 10^9^ /L
Monocytes	0.1	0.6	0.2-0.95	x 10^9^ /L
Basophils	0.1	0	0.0-0.1	x 10^9^ /L
ESR	5	-	1-10	mm
CRP	1.58	38.53	0-5	mg/L
Urea	4.4	5.1	2.5-7.8	mmol/L
Creatinine	72	62	64-104	µmol/L
Sodium	139	137	133-146	mmol/L
Potassium	4	4.2	3.5-5.3	mmol/L
Serology				
HCV-Ab	Negative			
HBsAg	Negative			
HIV 1 and 2	Negative			

Unfortunately, he was readmitted with worsening neurological symptoms on day 3. On examination, his GCS was 15/15, with diplopia in both eyes, reduced left-hand power (4/5), ataxia, and no other focal neurological deficits. MRI with contrast of the brain was normal, but the cerebrospinal fluid (CSF) examination indicated a slightly raised white cell count (52, predominantly lymphocytes at 90%), elevated protein (0.51 g/L, Normal 0.15-0.40 g/L), and normal glucose levels (3.7 mmol/L, Normal 2.22-3.89 mmol/L), with no organisms identified (Table 2). Remote neurology consultation suggested a probable autoimmune demyelinating illness, likely multiple sclerosis, and recommended high-dose steroids (intravenous methylprednisolone 1 g once a day for 3 days). However, due to a lack of clinical improvement and the development of a new fever, empirical antibiotics for central nervous system (CNS) infection were started on day 5. He received intravenous ceftriaxone 2 g twice a day, aciclovir 800 mg (10 mg/kg) three times a day, and dexamethasone 8.3 mg four times a day for meningoencephalitis. His condition further deteriorated on day 6 with new right-sided weakness, leading to his transfer to critical care. His GCS was 13/15 (E3V4M6), pupils constricted with slow light response, right facial droop, increased tone, dense left-sided weakness, neck rigidity, and a positive Babinski sign.

**Table 2:** Cerebrospinal fluid (CSF) analysis ESR: erythrocyte sedimentation rate; CRP: C-reactive protein; HCV AB: hepatitis C virus antibody; HBS AG: hepatitis B surface antigen; HIV: human immunodeficiency virus; AFB: acid-fast bacillus

CSF Investigation	Day 3	Day 6	Normal Values
CSF appearance	Slightly blood-stained	Clear	Clear
CSF pressure	20	50	10 - 25 cm H2O
CSF cytology red blood cells	156	24	0-5
CSF cytology white cell count (WCC)	52	1048	0-5
CSF WCC differential	90% Lymphocytes, 10% Polymorph	90% Lymphocytes, 10% Polymorphs	70% Lymphocytes, 30% monocytes
CSF glucose	3.7	6.7	2.22 – 3.89 mmol/L
CSF proteins	0.51	1.44	0.15 -0.40 g/L
CSF Lactate	-	6.0	0.6 -2.44 mmol/L
CSF IgG	40	145	10-45 mg/L
CSF albumin	296	785	10-45 mg/L
CSF: serum IgG/albumin ratio	0.7	0.9	0.2-0.7
CSF oligoclonal bands	Negative	Negative	Negative
CSF microscopy	No organism seen	No organism seen	No organism
CSF multiplex PCR	No pathogen in PCR	Listeria monocytogenes	Negative
CSF culture	No growth	No growth	Sterile
CSF Cryptococcal antigen	-	Negative	Negative
CSF tuberculosis (AFB, culture)	-	Negative	Sterile

**Figure 1:**
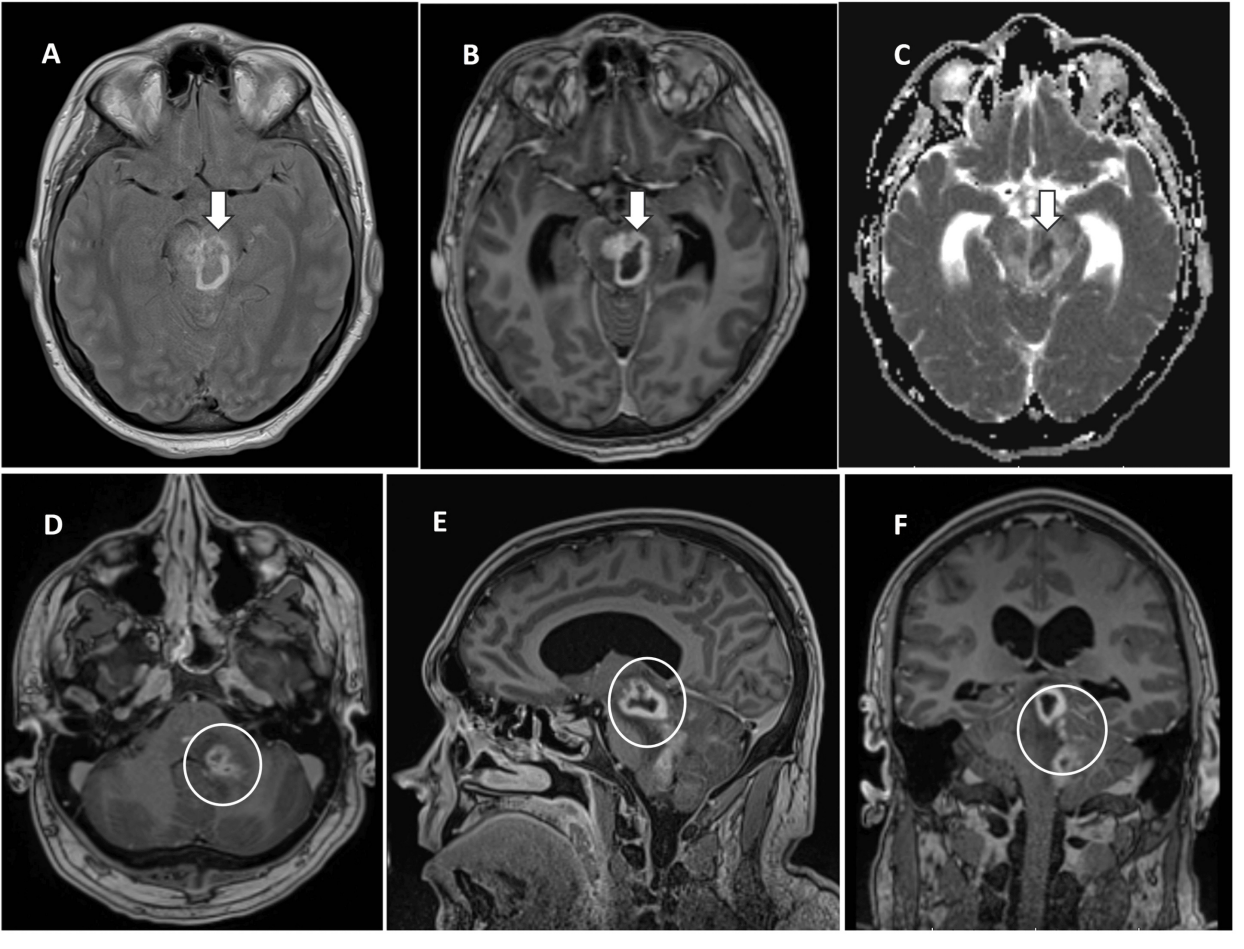
Brain magnetic resonance imaging on day 14 1A: T2-weighted imaging showing heterogeneous high signal in left midbrain (white arrow); 1B: post-contrast T1-weighted axial image demonstrating thin rim enhancement surrounding the lesion (white arrow); 1C: apparent diffusion coefficient (ADC) map showing central restricted diffusion, consistent with abscess formation (white arrow); 1D: T1-weighted axial post-contrast image showing scattered areas of enhancement within the pons and left cerebellar hemisphere (circle); 1E: T1-weighted sagittal post-contrast image showing the midbrain lesion (circle); 1F: T1-weighted coronal post-contrast image again demonstrating the left midbrain and cerebellar lesion (circle)

A repeat CSF examination showed an elevated opening pressure of 50 cm H2O and a significant rise in white cell count to 1048 (Normal 0-5), protein to 1.44 g/L (Normal 0.15-0.40), lactate to 6.0 mmol/L (Normal 0.6 -2.44), and glucose to 6.7 mmol/L (Normal 2.22-3.89) (Table 2). Later, *Listeria monocytogenes *was detected in the same CSF sample via multiplex polymerase chain reaction (PCR). However, there was no growth in culture. Ceftriaxone, aciclovir, and dexamethasone were discontinued (total of 2 doses of ceftriaxone and aciclovir and one dose of dexamethasone given), and intravenous Amoxicillin 2 g four hourly was commenced, on advice from the infectious disease (ID) team as per United Kingdom (UK) national guidelines on meningitis [4]. His GCS improved briefly to 14/15 (E4V4M5) after lumbar puncture, with persistent right-sided weakness. He was transferred to Neuro Critical care after discussion with neurology and ID team on day 7 with a GCS of 14/15. Unfortunately, overnight, his neurology worsened with GCS 7/15 (E1V3M3), and he was intubated. On day 8, the ID team switched his antibiotic to intravenous meropenem 2 g three times a day due to a drug rash from amoxicillin. Imaging revealed a midbrain abscess and obstructive hydrocephalus on day 12 (Figure 1). He underwent external ventricular drainage (EVD) and later a ventriculoperitoneal (VP) shunt. Adverse reactions to standard therapeutic antibiotic agents (drug rash from linezolid, amoxicillin, and trimethoprim-sulfamethoxazole) again complicated his stay.

**Figure 2:**
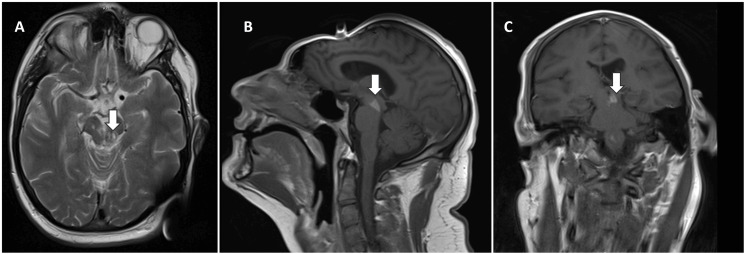
Brain magnetic resonance imaging after two months 2A: T2-weighted axial image showing reduced oedema and residual signal changes in the left midbrain; 2B: T1-weighted sagittal post-contrast image showing marked reduction of the previously noted lesion; 2C: T1-weighted coronal post-contrast image demonstrating only minimal residual enhancement

**Figure 3:**
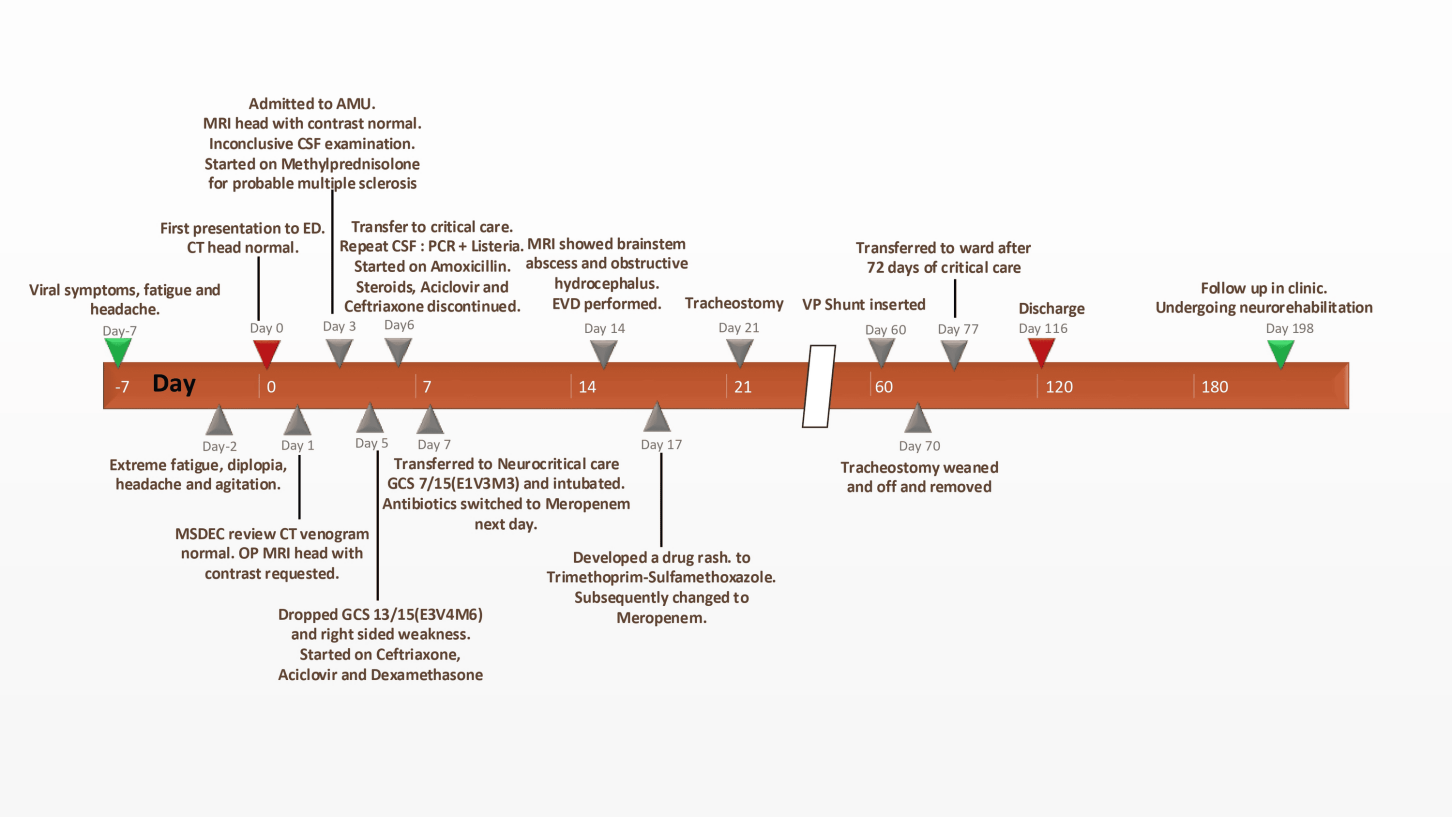
Timeline of clinical course ED: emergency department; CT: computed tomography; MSDEC: medical same Day emergency clinic; OP: outpatient; AMU: acute medical unit; MRI: magnetic resonance imaging; CSF: cerebrospinal fluid; GCS: Glasgow Coma Scale [3]; PCR: polymerase chain reaction; EVD: external ventricular drainage; VP: ventriculoperitoneal

Ultimately, he completed 12 weeks of meropenem. MRI with contrast of the brain at the end of the period revealed residual changes in the left brain, with no evidence of relapsed disease (Figure 2). He was discharged on day 116 to neurorehabilitation, with good recovery noted at three-month follow-up (summarised in Figure 3). He has normal cognition, diplopia that is improving with orthoptics, mild dysarthria, paraparesis with motor strength at 3/5, and presently requires assistance when standing and is on a wheelchair for mobility. He has been making slow but steady progress with physiotherapy and neurorehabilitation.

## Discussion

Neurolisteriosis is an invasive listerial disease characterised by meningeal and parenchymal involvement, often leading to high mortality and morbidity. Common manifestations include meningitis and meningoencephalitis, while rhombencephalitis and brain abscess are less frequent [2]. Individuals who are immunocompromised, pregnant, or older are at increased risk, although cases can occur in healthy young adults. The clinical features may differ from those of other bacterial meningitides, as it may have a subacute course [5,6]. It often presents with altered sensorium and fever, but with a lower incidence of meningeal signs [6].

In the presented case, the patient exhibited primarily neurological signs, normal inflammatory markers, and inconclusive CSF and imaging results that did not support a diagnosis of bacterial meningitis. Ruling out cerebrovascular and inflammatory demyelinating diseases pointed toward an infectious cause. CSF pleocytosis in listeriosis can manifest as either lymphocytic or neutrophilic [7]. United Kingdom (UK) national guidelines on meningitis recommend additional coverage for *Listeria *in patients aged 60 and above or those who are immunocompromised [4]. Therefore, empirical treatment for *Listeria* was not indicated. In this case, a subsequent CSF sample showed predominantly lymphocytes and elevated protein levels, and confirmed *Listeria* via multiplex PCR. However, CSF culture was negative. This highlights a major pitfall in the diagnosis of listeriosis, as it is difficult to culture from CSF. Therefore, advanced molecular diagnostics, such as PCR, are a vital diagnostic tool when suspicion is high.

Neurolisteriosis is typically treated with amoxicillin, ampicillin, or penicillin G, with alternatives such as trimethoprim-sulfamethoxazole or meropenem, usually administered for at least 21 days [4-6]. In the UK, national guidelines recommend amoxicillin for the treatment of* Listeria* [4]. However, in this case, a prolonged course of meropenem was chosen due to complications from a brain abscess requiring EVD and the adverse effects of other standard antibiotics.

The use of dexamethasone in bacterial meningitis is controversial, with some studies suggesting it may increase mortality in CNS listeriosis [8,9]. This case raises questions about why a healthy, immunocompetent individual developed neurolisteriosis, and, secondly, a florid presentation following treatment with high doses of steroids. Corticosteroid-induced lymphocyte redistribution and immunosuppression are well-recognised mechanisms [10]. We have a strong suspicion that high-dose steroid administration in the initial presentation may have contributed to the rapid deterioration because of transient immunosuppression. The mortality rates from neurolisteriosis range from 15% to 36%, with significant neurologic sequelae in 55% survivors [7]. According to the 2017 MONALISA study, the presence of concurrent bacteremia, active cancer, multi-organ failure, and adjunctive use of dexamethasone was associated with significantly reduced survival [9]. The prolonged recovery of our patient aligns with the high morbidity observed in the MONALISA study. This case also highlights the variability in the presentation of neurolisteriosis and the crucial need to consider differentials for CNS infection, even in immunocompetent individuals.

## Conclusions

Neurolisteriosis remains a challenging health problem because of high mortality and morbidity rates. Diagnosing listeriosis can be difficult, as cerebrospinal fluid (CSF) analysis may yield inconclusive results, including negative cultures. *Listeria monocytogenes *should be considered a key differential diagnosis in any patient who fails to respond to standard antibiotic treatment for bacterial meningitis. Early diagnosis and treatment can significantly improve patient outcomes and reduce mortality rates.
